# The Expression of *TGF-β1*, *SMAD3*, *ILK* and miRNA-21 in the Ectopic and Eutopic Endometrium of Women with Endometriosis

**DOI:** 10.3390/ijms24032453

**Published:** 2023-01-26

**Authors:** Anna Zubrzycka, Monika Migdalska-Sęk, Sławomir Jędrzejczyk, Ewa Brzeziańska-Lasota

**Affiliations:** 1 Department of Biomedicine and Genetics, Medical University of Lodz, 92-213 Lodz, Poland; 2 Operative and Conservative Gynecology Ward, Dr K. Jonscher Municipal Medical Centre, 93-113 Lodz, Poland; 3 Institute of Medical Expertises, 91-205 Lodz, Poland

**Keywords:** endometriosis, expression genes, *TGF-β1*, *SMAD3*, *ILK*, miR-21, EMT

## Abstract

The molecular pathogenesis of endometriosis has been associated with pathological alterations of protein expression via disturbances in homeostatic genes, miRNA expression profiles, and signaling pathways that play an essential role in the epithelial-mesenchymal transition (EMT) process. *TGF-β1* has been hypothesized to play a key role in the development and progression of endometriosis, but the activation of a specific mechanism via the TGF-β-SMAD-ILK axis in the formation of endometriotic lesions is poorly understood. The aim of this study was to assess the expression of EMT markers (*TGF-β1*, *SMAD3*, *ILK*) and miR-21 in ectopic endometrium (ECE), in its eutopic (EUE) counterpart, and in the endometrium of healthy women. The expression level of the tested genes and miRNA was also evaluated in peripheral blood mononuclear cells (PBMC) in women with and without endometriosis. Fifty-four patients (*n* = 54; with endometriosis, *n* = 29, and without endometriosis, *n* = 25) were enrolled in the study. The expression levels (RQ) of the studied genes and miRNA were evaluated using qPCR. Endometriosis patients manifested higher *TGF-β1*, *SMAD3*, and *ILK* expression levels in the eutopic endometrium and a decreased expression level in the ectopic lesions in relation to control tissue. Compared to the endometrium of healthy participants, miR-21 expression levels did not change in the eutopic endometrium of women with endometriosis, but the RQ was higher in their endometrial implants. In PBMC, negative correlations were found between the expression level of miR-21 and the studied genes, with the strongest statistically significant correlation observed between miR-21 and *TGF-β1*. Our results suggest the loss of the endometrial epithelial phenotype defined by the differential expression of the *TGF-β1*, *SMAD3* and *ILK* genes in the eutopic and ectopic endometrium. We concluded that the TGF-β1-SMAD3-ILK signaling pathway, probably via a mechanism related to the EMT, may be important in the pathogenesis of endometriosis. We also identified miR-21 as a possible inhibitor of this TGF-β1-SMAD3-ILK axis.

## 1. Introduction

Endometriosis is a debilitating, estrogen-dependent chronic gynecological disease, which is characterized by the presence of endometrial-like tissue and the growth of dysfunctional endometrial glands and stroma outside the uterine cavity [1]. The incidence of endometriosis is difficult to estimate, as some patients with this pathology are asymptomatic; therefore, as much as 11% of cases remain undiagnosed [2,3]. The prevalence rate of clinically symptomatic endometriosis that involves dysmenorrhea, dyspareunia, dysuria, dyschezia, infertility, and cyclic and acyclic pelvic pain [4,5] among women of reproductive age worldwide is estimated at 10–15% [3,6]. Although spontaneous remission is possible, it is suggested that endometriosis with moderate and severe atypia may affect cell architecture and proliferation, resulting in a possible malignant transformation [6,7]. In particular, ovarian endometriosis—one of the most common subtypes occurring in 17–44% of women diagnosed with endometriosis [8] is considered to possess a premalignant potential, which supports a model of endometriosis as a precursor of malignant ovarian cancer. The progression of endometriosis to ovarian cancer has been supported by molecular evidence. The pathogenesis of both diseases consists of cell invasion, unrestrained growth, the development of new blood vessels, and decreased apoptosis and inflammatory responses [7].

At the molecular level, these changes are often associated with pathological protein expression via disturbances in homeostatic genes as well as miRNA expression profiles and signaling pathways involved in ovarian carcinogenesis and, similarly, in endometriosis development. For example, the overexpression of p53, pro-inflammatory cytokines such as: transforming growth factor (TGF)-β1, cyclooxygenase (COX)-2, vascular endothelial growth factor (VEGF) and tumor necrosis factor-alpha (TNF-α), as well as estrogen (ER)-1α and androgen (AR) receptors, or decreased expression levels of the progesterone receptor (PR) and inflammatory interleukin 1 (IL-1), were revealed. All of them presented a similar expression pattern in both ovarian endometriosis tissues and endometriosis-associated ovarian cancer [9,10,11].

Given the reasonable assumptions and also the evidence that endometriosis may pose a risk of malignancy depending on changes in the expression of important genes and regulatory miRNAs, it seems important to provide new information on the molecular origin and development of endometriosis itself. To date, the cause of this disease remains not fully elucidated, and its pathogenesis is only partially understood. Recent studies suggest that mechanisms related to the epithelial-mesenchymal transition (EMT) may play a crucial role in endometriosis. EMT is associated with the disruption of signal transduction pathways and the loss of polarity and intercellular contacts in the epithelial and mesenchymal cells. During this multi-stage process, immotile epithelial cells acquire phenotypes of motile mesenchymal cells and the ability to migrate and invade other regions, which is accompanied by changes in cell morphology and gene expression [12,13].

The transforming growth factor-β1/Smad3 (TGF-β1/Smad3) signaling pathway seems to play an essential role in the EMT mechanism. TGF-β has been shown to be an inhibitor of the proliferation of normal endometrial cells, arresting the cell cycle in the G1 phase. As the major promoter of EMT, it is differentially and abundantly expressed in the endometrium under hormonal control [14,15]. *TGF-β* up-regulation in the endometriotic tissue, serum, and peritoneal fluid of endometriosis patients may be crucial for the development and/or maintenance of this disease [16,17,18]. Moreover, TGF-β1 is recognized as an important profibrogenic mediator, and probably along with another strong fibrogenesis stimulus, platelet-derived growth factor (PDGF), it can induce increased collagen production, cell contractility, and metaplasia of the endometrial epithelium, which ultimately leads to fibrosis. Such a phenotypic transformation has often been found in ovarian, peritoneal, pleural and extra-organ endometriosis [19,20,21].

The published literature emphasizes the role of activated Smad2 and Smad3 in collagen expression and tissue fibrosis that operate downstream of TGF-β1 ligands [22,23]. Acting as transcription factors, the Smads (Smad2, p-Smad2, Smad3, p-Smad3, Smad2/3, and Smad4) are the key elements of the TGF-β/Smad signaling pathway. Smads are positive in the endometriotic cystic wall and also active in the peritoneum of patients with endometriosis [18,23]. In contrast, the lower number of immunopositive Smad cases shown in ovarian endometriosis than in normal endometrium suggests a suppressive effect on the proliferation of endometrial cells, with the simultaneous disturbance of Smad expression during the formation of endometrial lesions or the involvement of non-Smad signaling pathways in ectopic endometrial cells [24,25]. The Smad protein family ultimately regulates the transcription of genes such as E12/E42, ZEB1, ZEB2, Snail1, Snail2/Slug, and Twist1, resulting in the loss of cell–cell tight and adherens junctions, desmosomes, and the gain of mesenchymal markers characteristic of EMT [16].

Existing evidence has demonstrated the TGFβ-1 regulation of EMT to also be dependent on integrin-linked kinase (ILK) function during the fibrosis process [26,27]. This serine/threonine protein kinase is a focal adhesion adapter, mediating the cell–extracellular matrix (ECM) and cell–cell relationships [28]. Its fundamental role is to regulate cell survival, proliferation and migration by connecting the cytoplasmic domains of β-integrins with the actin cytoskeleton, mediating integrin signaling in various cells [27,29]. Data presented in recent studies broaden this observation by showing that overexpression of *ILK* increases the ability of human endometrial stromal cells to migrate and invade by facilitating EMT [30]. It is also proposed that the regulation of this process may be associated with ILK-Akt [31] and ILK/GSK-3β/β-catenin/slug signaling pathways [32]. The cyclic switching of integrins is known to take place in endometrial cells throughout the menstrual cycle, and since ILK acts at the central stage of cell-matrix adhesion and can induce focal adhesions, it is reasonable to assume that ILK may also play an important role in the unique pathology of the ectopic spread of the endometrium [33]. While there is agreement that ILK is biologically important, especially in the context of EMT, the expression profile of this gene in endometriotic lesions has yet to be determined. Extensive research into the understanding of endometriosis and its evolution toward ovarian cancer has identified a number of miRNAs with dysregulated expression patterns. Among the range of miRNAs (miR-1, miR-17, miR-30a, miR-34, miR-141, miR-145, miR-155, miR-200a/b/c, miR-205, miR-325 and miR-429) that may contribute to neoplastic transformation, miR-21 is also mentioned [6,12]. It is the first oncogenic miRNA (oncomir) that is commonly expressed in many human cancers, including ovarian, breast, thyroid, colon, rectal, prostate, lung and pancreatic carcinomas [34]. MiR-21 is also dysregulated in endometrial cancer and participates in EMT [35]. It plays an important role in cell proliferation, migration, apoptosis, and invasion and is clearly involved in the key regulatory pathways. Recent studies show that the modulation of its expression results in clear and significant phenotypic changes in endometrial epithelial cells [36,37]. All the above-mentioned processes in which miR-21 participates play an important role in the development and progression of endometriosis; however, the involvement of miR-21 in this disease entity is little studied. miRTarBase suggests that many genes are potential targets of miR-21-5p. This database also points to a miR-21-5p–*TGF-B1* interaction, thus speculating a regulatory function of miR-21-5p in modulating *TGF-β1* expression (https://mirtarbase.cuhk.edu.cn).

Taken together, the expression and activation of EMT-inducing factors occur in response to a variety of signaling pathways, including those mediated by *TGF-β1*, *SMAD3*, and *ILK* signaling, which are the objects in this work. We assume that some of these signals may be predominant in targeting EMTs at different stages of reprogramming, which may result in differentiated expression of genes depending on the microenvironment, tissue type, and endometriotic lesion location. More importantly, disturbances in intracellular signaling pathways related to the pathological expression of EMT mesenchymal biomarkers (*TGF-β1*, *SMAD3* and *ILK*) may have clinical significance in the diagnostic and therapeutic aspects of endometriosis. There is still a need to search for early and non-invasive diagnostic markers related to the process of primary changes inside the endometrium itself, changes related to metaplasia and to EMT. Therefore, in order to assess the potential dysregulation of expression in ectopic endometrium, the expression profiles of *TGF-β1*, *SMAD3*, *ILK* and miR-21 were compared not only with their eutopic counterparts but also with the level of expression in the endometrium of healthy women. The present study also determines the expression level of the studied genes and miRNAs in PBMC in women with and without endometriosis. Additionally, the purpose of the study was to analyze the correlation between *TGF-β1*, *SMAD3*, *ILK* and miR-21 expression levels and the clinical features and biochemical parameters of patients with endometriosis.

## 2. Results

### 2.1. The Expression Profile of TGF-ß1, SMAD3, ILK and miR-21 in Ectopic Lesions (ECE), Eutopic Endometrium (EUE) vs. Control Endometrium (C1)

mRNA *TGF-ß1*, *SMAD3*, *ILK* and miR-21 were expressed in all evaluated tissues. The expression level in ectopic lesions and matched eutopic endometrium obtained from the same patient and in control tissues were compared. In patients with endometriosis, the mean *TGF-ß1* gene expression level (RQ value) was 0.317748 in the ECE and 2.760536 in the EUE, while in the endometrium of the control women, the mean RQ was 0.654076. In the case of *SMAD3*, the mean RQ was 0.318956 in the ECE and 0.902240 in the EUE of patients with endometriosis, and 0.543976 in the endometrium of the control group. For *ILK*, the RQ value was 0.357348 in the ECE and 7.755932 in the EUE of patients with endometriosis, while in the endometrium of women without endometriosis (C1), the mean RQ value was 2.524812. In patients with endometriosis, the mean expression level (RQ value) of miR-21 was 17.58223 in ECE and 0.97790 in EUE, while in the endometrium of control women, the mean RQ was 1.165748.

Kruskal–Wallis rank analysis demonstrated a statistically insignificant difference in the expression levels of the *TGF-ß1* (*p* = 0.0601) and *SMAD3* (*p* = 0.0701) genes between the studied groups. In the case of *ILK* and miR-21, the Kruskal–Wallis rank analysis showed a statistically significant difference in the level of expression between the studied groups (*p* = 0.0075 and *p* = 0.00001, respectively). The Newman-Keuls post hoc test used suggested a negligible trend towards lower expression in ECE than in EUE for *TGF-ß1* (*p* = 0.070437) as well as *SMAD3* (*p* = 0.060457) and higher expression in EUE compared to C1 for *TGF-ß1* (*p* = 0.056815). In contrast, the expression level of miR-21 was significantly higher in ECE than in EUE (*p* = 0.000116) and in ECE compared to C1 (*p* = 0.000117; Newman–Keuls test). The above differences for miR-21 were confirmed by an additional Mann–Whitney U test (*p* = 0.0000001). The additional Mann–Whitney U test also showed statistically significantly lower expression of *TGF-ß1*, *SMAD3* and *ILK* in ectopic lesions compared to matched eutopic endometrium in women with endometriosis (*p* = 0.023471, *p* = 0.026057 and *p* = 0.006639; respectively). In addition, *ILK* expression levels were significantly lower in ectopic lesions compared to the control (Mann–Whitney U test, *p* = 0.006236).

**Figure 1:**
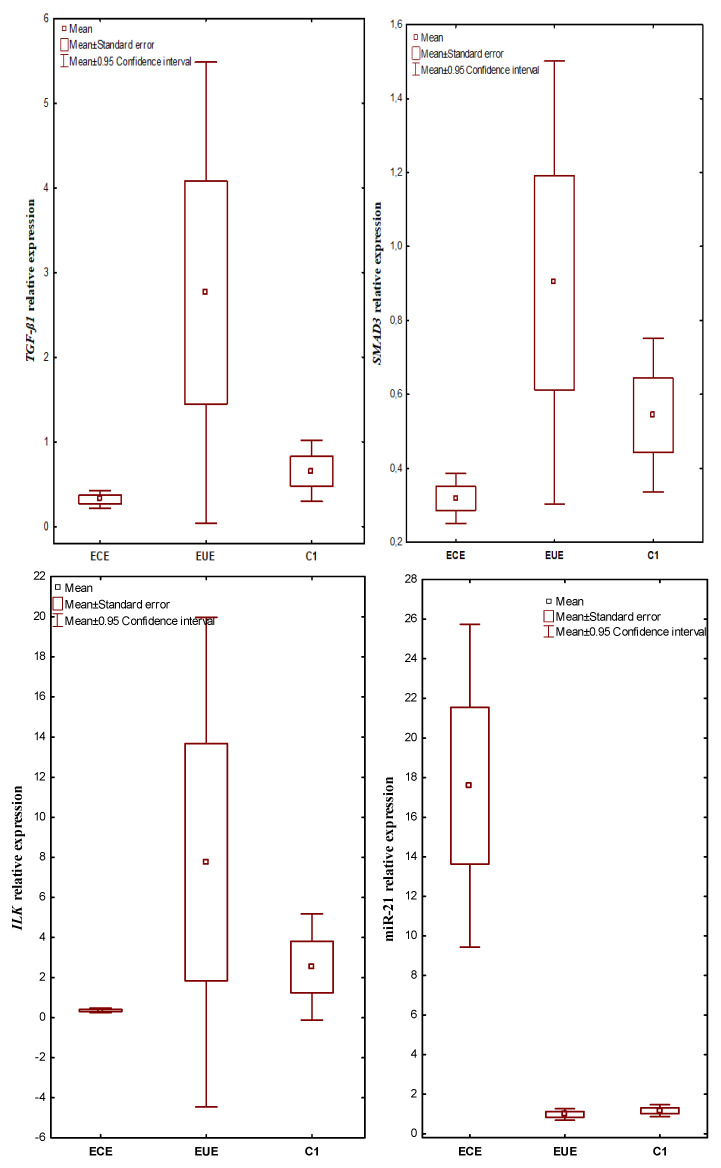
The expression levels (mean RQ values) of the studied genes and miR-21 in individual tissue materials (sources of tissue: ectopic endometrium—ECE and eutopic endometrium—EUE, obtained from the same patient with endometriosis) and control endometrium (C1).

The results of expression levels (mean RQ values) of the studied genes and miR-21 in individual tissue materials (ectopic endometrium—ECE and eutopic endometrium—EUE, obtained from the same patent with endometriosis) and control endometrium (C1) are presented in Figure 1.

### 2.2. The Expression Profile of TGF-ß1, SMAD3, ILK and miR-21 in PBMCs: From Patients with Endometriosis vs. from Patients without Endometriosis (C2)

The expression levels of the *TGF-ß1, SMAD3, ILK* and miR-21 genes were assessed in PBMCs obtained from patients with endometriosis and in PBMCs from patients without endometriosis, who constituted the control group (C2). No statistically significant differences in the level of expression of the genes and miR-21 in PBMCs were observed between the studied groups (*p* > 0.05, Mann–Whitney U test). The results are presented in Table 1.

### 2.3. Correlations between the Expression Level of TGF-ß1, SMAD3, ILK and miR-21 in 3 Different Biological Materials (Ectopic Endometrium–ECE, Eutopic Endometrium–EUE, and PBMCs) Obtained from the Same Patient with Endometriosis

In the ectopic endometrium, a positive correlation was demonstrated between the *ILK* and *SMAD3* genes (Rho = 0.496154, *p* = 0.011652; Spearman’s rank correlation) as well as *ILK* and *TGF-ß1* (Rho = 0.649231, *p* = 0.000446; Spearman’s rank correlation).

Similarly, in the eutopic endometrium, a positive correlation was found between the *ILK* and *SMAD3* genes (Rho = 0.904615, *p* = 0.0000001; Spearman’s rank correlation) as well as *ILK* and *TGF-ß1* (Rho = 0.913077, *p* = 0.0000001; Spearman’s rank correlation). In addition, a positive correlation was observed between the *TGF-ß1* and *SMAD3* genes (Rho = 0.830000, *p* = 0.0000001; Spearman’s rank correlation).

**Figure 2:**
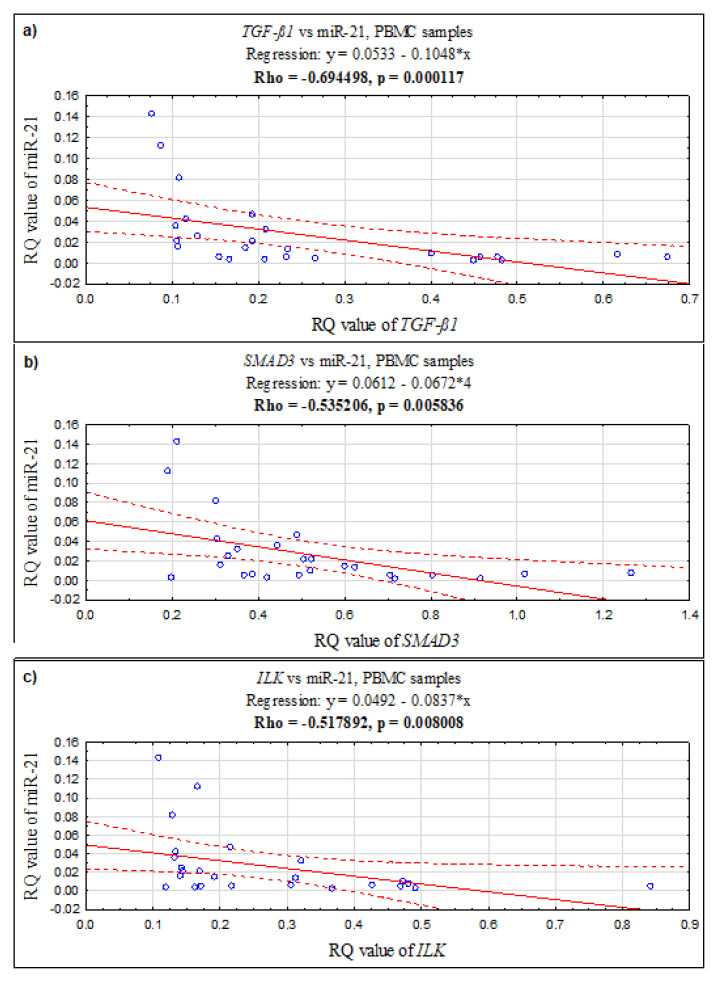
The scatter plots showing correlations between expression levels (RQ values) of miR-21 and genes: (**a**) *TGF-ß1*, (**b**) *SMAD3*, (**c**) *ILK* in PBMC samples.

In PBMCs, a positive correlation was found between the *ILK* and *SMAD3* genes (Rho = 0.726923, *p* = 0.000039; Spearman’s rank correlation), *ILK* and *TGF-ß1* (Rho = 0.870769, *p* = 0.0000001; Spearman’s rank correlation), and *TGF-ß1* and *SMAD3* (Rho = 0.683846, *p* = 0.000164; Spearman’s rank correlation). Additionally, in PBMCs, the expression level of miR-21 negatively correlates with *TGF-ß1* (Rho = −0.694498, *p* = 0.000117; Spearman’s rank correlation), with *SMAD3* (Rho = −0.535206, *p* = 0.005836; Spearman’s rank correlation), and with *ILK* (Rho = −0.517892, *p* = 0.008008; Spearman’s rank correlation) (see Figure 2).

### 2.4. Correlations between the Expression Level of TGF-ß1, SMAD3, ILK, and miR-21 in Relation to Clinical Characteristics and Biochemical Parameters of Patients with Endometriosis

The RQ values for the TGF-ß1, SMAD3, and ILK genes, as well as miR-21, in ectopic tissue were analyzed in relation to the clinical features of patients with endometriosis: age at the time of diagnosis, stage of endometriosis (according to rASRM classifications), pelvic pain symptoms (according to the numerical rating scale–NRS), phase of the menstrual cycle, and concentrations of the biochemical parameters of CA-125 and HE4. The results are shown in Table 2.

Analysis of the level of expression of the tested genes and miRNA-21 in the group of patients with endometriosis according to age showed that the expression of all the studied genes was higher in patients aged ≤40 years compared to older patients, whereas miR-21 expression was higher in patients >40 years of age, although the observed differences were not statistically significant (*p* > 0.05).

In tissue material collected from patients in the proliferative phase of the cycle, the level of expression of the tested genes was higher compared to tissue samples from patients in their secretory phase. MiR-21 expression was higher in tissue samples from patients in the secretory phase of the cycle, but the changes in expression were not statistically significant (*p* > 0.05, Mann–Whitney U test). There were also no statistically significant differences in the level of expression of the studied genes and miR-21 between the proliferative and the early, middle and late secretory phases of the menstrual cycle (*p* > 0.05, Kruskal–Wallis test).

Analysis of the level of gene and miR-21 expression according to the stage of endometriosis demonstrated a statistically insignificant increase in the level of expression of *TGF-ß1* and miR-21 in the group of patients with stage IV compared to stage III of endometriosis (*p* >0.05, Mann–Whitney U test). An inverse correlation was observed between the level of expression of the *SMAD3* and *ILK* genes and the stage of the disease; however, in the case of the *ILK* gene, only a negligible downward trend in *ILK* expression was observed in the group of patients with stage IV vs. stage III of endometriosis (*p* = 0.086857, Mann–Whitney U test).

Analysis of the level of gene and miR-21 expression according to the severity of pain showed a decrease in the level of expression of *TGF-ß1* and miR-21 in the group of patients with very strong painful symptoms occurring in the course of endometriosis, but the changes in expression were not statistically significant (*p* > 0.05, Mann–Whitney U test). In contrast, the level of expression of the *SMAD3* and *ILK* genes correlated positively with the degree of pain severity, and for the *ILK* gene, a statistically significant increase in expression was confirmed in the group of patients with very strong painful symptoms (*p* = 0.044271, Mann–Whitney U test), while for the *SMAD3* gene, only a negligible upward trend in expression was observed in that group of patients (*p* = 0.071229, Mann–Whitney U test).

By dividing the group of patients with endometriosis according to the concentration of Ca 125 into the ≤65 U/mL vs. >65 U/mL subgroups, a statistically insignificant increase in the level of expression of the tested genes in the group of patients with a higher concentration of Ca 125 was confirmed, whereas the expression of miR-21 was higher in the group of patients with Ca 125 ≤65 U/mL (*p* > 0.05, Mann–Whitney U test). Likewise, a statistically insignificant increase in the gene expression correlated with higher HE4 concentrations, while the level of miRNA expression was lower in that group of patients (*p* >0.05, Mann–Whitney U test).

## 3. Discussion

The pathophysiology responsible for the onset, development, and course of endometriosis progression remains unsolved. The lack of clinically relevant biomarkers with requisite sensitive and specificity means that the average lag phase between the onset of symptoms of endometriosis and its diagnosis has been estimated to be up to 8–12 years [38,39]. Therefore, the major priority of endometriosis research is the discovery of a non-invasive biomarker or a panel of such biomarkers that would hasten the diagnosis of this disease in women who have pelvic pain and/or subfertility with normal ultrasound results [40]. This is essential due to the fact that endometriosis in a significant number of women has long-term progress without clear symptoms of the disease; hence, the diagnostic process is very long [38,39].

Accumulating evidence on the pathogenesis of endometriosis indicates a polygenetic cause of this disease, but none of the candidate genes have yet been approved as a molecular marker useful for the clinical diagnosis of gynecologic tumors, including endometriosis [1,3,41]. Moreover, despite intensive research on the role of miRNAs which showed changes in expression patterns in endometrial stromal cells, the data on its role in the regulation of pathogenic processes related to the formation of endometrial lesions are still inconsistent [6,11,42].

In connection with the above, in our study, we evaluated the expression of EMT markers (TGF-β1, SMAD3, ILK) and miR-21 in ectopic endometrium (ECE), its eutopic portion (EUE), and in the endometrium of healthy women. Peripheral blood mononuclear cells (PBMCs) from endometriosis patients and non-patients were also investigated.

We suggest that the endometrial epithelial phenotype is defined by the differential expression of TGF-β1, SMAD3, and ILK genes between ectopic and eutopic endometrium. Furthermore, we conclude that the TGF-β1-SMAD3-ILK signaling pathway may be important in the etiology of endometriosis, possibly via EMT-related mechanisms, and we identify miR-21 as an inhibitor of this TGF-β1-SMAD3-ILK axis.

TGF-β1, as a multifunctional cytokine, is thought to play an important role in the development and progression of endometriosis. It was confirmed that at the mRNA level, *TGF-β* regulates in the endometrium the expression of the extracellular matrix, adhesive molecules, and proteases. Its altered expression is associated with the manifestation of pathological uterine functions, including endometriosis [43]. Activated TGF-β receptors induce the recruitment of multiple specific intracellular signals, including SMADs, which can accumulate in the nucleus and possess the function of transcription factors to modulate the expression of genes and miR-21 after phosphorylation [44,45]. At the same time, many elements of ILK signaling are induced by *TGF-β* in a *SMAD*-dependent manner [46]. The specific mechanism of the TGF-β-SMAD-ILK axis in the pathology of endometriosis has been poorly studied. Research evidence suggests that the establishment of endometriosis may be facilitated by endometrial dysfunction involving both eutopic and ectopic endometrium [47].

In our study, changes in the expression levels of miR-21, *TGF-β1*, *SMAD3* and *ILK* were assessed in both tissues. In addition, we examined the mRNA levels of the genes and miRNA considered in peripheral blood mononuclear cells (PBMC) of patients with and without endometriosis in order to search for informative diagnostic biomarkers. As one of the findings of our study, we detected significantly reduced *TGF-β1* expression in the ectopic endometrium compared to eutopic tissue in women with endometriosis. Interestingly, most research focuses on understanding the physiological role of this gene in the human endometrium, emphasizing that *TGF-βs* are synthesized in the endometrium in large quantities and promote its adhesion to ovarian tissue [23,48,49]. Moreover, *TGF-β* expression is under hormonal control, and its expression is specific to the endometrial stage [15]. The highest level of *TGF-β1* expression is observed during menstruation [50]. It has been suggested that *TGF-β1* expression may play a role in the mechanisms regulating the transition from proliferation through invasion and motility to the differentiation of endometrial cells [51]. Thus, this pleiotropic cytokine, by participating in a number of important processes during endometrial tissue remodeling in the perimenstrual period, may promote endometrial tissue repair and protect from extensive fibrosis and scarring [52]. In view of the above data, our observation of high *TGF-β1* expression in the eutopic endometrium confirms its protective role and participation in endometrial tissue remodeling. Our observations and conclusions are consistent with the results of Goteri et al. [53]. Another main finding of our study was the observation that the *TGF-β1* expression level in the ectopic endometrium was low. We conclude that it may be one of the molecular changes contributing to the progression of already implanted endometrial foci. In contrast, our results differ from those obtained by a team of other authors [54,55], where upregulation of *TGF-β1* in ectopic endometrium was demonstrated. Taking our result into consideration, the decreased expression of *TGF-β1* in ectopic endometrium may suggest the occurrence of possible post-transcriptional modulations of *TGF-β1* depending on the diverse microenvironment of the endometrium. The discrepancy in the results should not be surprising because the mechanisms involved in endometriosis in eutopic and ectopic locations can be different and conditioned by the tissue environment, which is likely to affect *TGF-β1* expression. Interestingly, other researchers have proven that the sites adjacent to peritoneal lesions have higher levels of *TGF-β1* mRNA transcripts than the distal sites [18], and the activity of *TGF-β1* is elevated at the sites affected by the disease [56].

In the presented study, we compared the level of *TGF-β1* expression in the eutopic endometrium of women with endometriosis and the control endometrium. The available evidence indicates the existence of physiological differences in normal endometrium compared to the eutopic endometrium of women with endometriosis. In the latter, there was a significant increase in endometrial cell survival as well as changes in DNA fragmentation and expression of *TGF-β1*, a factor involved in cell proliferation, differentiation and induction of programmed cell death [57]. We have also observed a change in *TGF-β1* expression in our study, where the level of *TGF-β1* mRNA was higher in the endometriosis-affected patients compared to the endometrium of patients without endometriosis, although that difference did not reach statistical significance. It should be pointed out that our result has been confirmed by other authors, who observed overexpression of *TGF-β1* mRNA in the eutopic endometrium compared to control tissue [53,58].

Interestingly, it has been noted that *TGF-β1* expression is cyclical in normal endometrium and observed mainly during the middle and late secretory phases, while the cyclic effect was not present in eutopic endometrium in women with endometriosis [57]. On the contrary, in our study, the level of expression of *TGF-β1* did not change throughout the menstrual cycle in the eutopic tissue of women without and with endometriosis, nor in endometrial foci. We conclude that such controversial research results may be due to the number of study groups of patients in the individual phases of the menstrual cycle and the heterogeneity of the cellular/tissue material used in the experiment because *TGF-β1* is known to be localized in stromal cells, glandular cells and endometrial macrophages [43]. However, the important result of our research is similar to other authors’ [43,57] unequivocal confirmation of the local changes in *TGF-β1* synthesis in patients with endometriosis, which may favor cell proliferation and survival in the pathological endometrial tissue.

Endometriotic cells have been proven to synthesize *TGF-β1*, inducing fibrosis around the endometrial foci. In this pathological process, which results in damage to the adjacent ovarian tissues, SMAD transcription factors are involved [23]. In our work, we also investigated the expression of *SMAD3* mRNA in the endometrium of women with endometriosis. The expression level of this gene was significantly lower in ECE compared to the matched EUE of women with endometriosis and also lower than in the controls. Similarly to our results, other authors also demonstrated reduced *SMAD3* transcript levels in sick women vs. controls, but in the cumulus cells of the antral [59]. In contrast, observations conducted in a group of patients with endometriosis, from whom the peritoneal adhesion biopsies were taken, show results opposite to ours [60]. However, it should be emphasized that our biological material consisted mainly of tissue fragments from endometrial ovarian cysts, which may explain the discrepancy in the results obtained. Nevertheless, as *SMAD3* is one of the most important intracellular mediators in TGF-β signaling [23], based on our observations, it can be hypothesized that in the case of a disorder of its expression in endometriosis, the functionality of all proteins in the *TGF-β* superfamily can be changed.

Contrary to our observations, Cruz et al. [61] showed a significantly reduced expression of *SMAD3* mRNA as well as the total SMAD3 protein in the eutopic endometrium (EUE) of women with endometriosis. However, because pSMAD3 levels were similar in patients with and without endometriosis, the authors postulate that the phosphorylation of SMAD3 is precisely regulated by specific mechanisms, mainly by activating TGFβ/activin receptor ligands [61]. Unfortunately, we have not studied these mechanisms. Analyzing the expression of *SMAD3* mRNA in the eutopic tissue of women with and without endometriosis, we also did not demonstrate statistically significant differences between the studied groups. The above result suggests that the endometrium of women with endometriosis is not subject to significantly abnormal activation of the SMAD protein cascade, and small changes in *SMAD3* mRNA expression are likely to be compensated further down the TGF-β/SMAD signaling pathway, which may, in effect, prevent malignant transformation. On the other hand, the system of TGF-β/SMAD activity regulation in the endometrium is complicated due to a number of receptors with serine-threonine kinases activity and the cooperation of various cytokines interacting with this pathway, which requires deeper investigation in the context of the development of endometriosis.

The TGF-β/SMAD pathway is integrated into the intracellular signaling network through cross-talk with other signaling pathways, such as the PI3K-Akt pathway, an important mediator of TGF-β-induced activation of various EMT responses [62,63]. The integrin-linked kinase (ILK) has also been reported to be involved in Akt activation by TGF-β [33], and by binding to intracellular integrins, it promotes signaling associated with cell adhesion, apoptosis, proliferation, or cell migration [64].

Elevated ILK expression has been detected in various human cancers originating from epithelial cells [65,66,67]. In the course of endometriosis, both eutopic and ectopic endometrial stromal cells exhibit adhesive properties with similarity to cancer cells, among others, through different expressions of peritoneal mesothelial adhesion factors, including loss of close connections with the consequent acquisition of invasive and metastatic properties [68]. As the initial stage of endometriosis is considered to be the adhesion ability of the exfoliated endometrial tissues retreating into the pelvic cavity, research also focuses on the specific adhesive particles, such as *ILK*, regulating the ability of endometrial stromal cells to adhere [30,31,69,70]. In our study, an increase in the level of *ILK* expression in the endometrium (EUE) of women with endometriosis compared to the control group indicates the importance of *ILK* in the pathogenesis of endometriosis. The research of other authors, where increased levels of mRNA *ILK* expression in endometrial tissues have been demonstrated, both eutopic and ectopic [30,31,69,71], is consistent with ours.

Interestingly, in our study, the *ILK* gene expression profiles in eutopic and ectopic tissue in endometriosis patients differed, manifesting the upregulation of *ILK* in the eutopic endometrium and downregulation in endometrial lesions compared to normal endometrium. The above result may suggest a loss of endometrial epithelial phenotype defined by a differential *ILK* expression in the ectopic and eutopic endometrium, and the likely underlying mechanism may include EMT via the TGF-β1-SMAD-ILK signaling pathway. Thus far, it has been demonstrated that ILK is a critical intracellular mediator of tubular EMT involved in the pathogenesis of chronic renal fibrosis [72]. In view of the fact that fibrosis is a characteristic feature of endometriosis and the expression of *ILK* in renal tubular epithelial cells is strictly controlled by *TGF-β1* and dependent on intracellular *SMAD* signaling [46,72], we speculate that an analogous molecular mechanism could occur in endometrial epithelial cells and promote their mesenchymal transformation in endometriosis. The strength of our results is supportive of the presence of an *ILK* connection with the extensive TGFß-SMAD signaling network, also possible in the endometrium. We present the result concerning a strong positive correlation between the expression levels of *ILK* and *SMAD3* as well as *ILK* and *TGF-ß1*. Moreover, the evidence of published studies indicates that *ILK* increases the ability of cells to migrate and invade through EMT induction, which plays a significant part in the initial formation of endometriosis [30,31,69,70]. Therefore, inhibition of the expression and activity of *ILK* is being increasingly considered as a new target in the prevention of endometriosis and a possible therapeutic alternative to hormone therapy, often effective but carrying numerous side effects [30,31,69,70].

An interesting result of our work is an increase in *ILK* expression along with an increase in pain severity in women with endometriosis. The mechanisms associated with the pathophysiology of the development of pelvic pain in these women are poorly understood. It has been speculated that the predominance of the inflammatory environment with the reaction of fibrosis and the formation of local scars and adhesions may be involved in this process. It is probable that the increased local release of pro-inflammatory cytokines in the endometrial tissue leads to excessive sensory innervation by impairing the regulation of neurotrophins [73]. Currently, there are no available data on the role of *ILK* in the development of chronic pelvic pain. *TGF-ß1* is known to affect the expression of growth factor (NGF) and neurotrophin-3 (NT-3) mRNA [74], and as *ILK* remains under *TGF-ß1* control [72], the involvement of *ILK* in the mechanism of pelvic pain in women with endometriosis is therefore possible.

More and more evidence indicates that altered miRNA expression may be involved in the development of endometriosis [11,75,76]. In several studies [77,78,79], including our own research work, the possible involvement of miR-21 in the pathogenesis of this disease has been emphasized. In the ectopic endometrial tissue (ECE) compared to the eutopic endometrium (EUE) in the same patient, we noted a significant increase in the level of miR-21 expression. Other authors have also observed that miR-21 exhibited an overexpression profile in endometriosis (in ECE) compared to paired eutopic endometrium [77,80]. Therefore, it seems that the altered expression of miR-21 may be relevant to the formation of endometrial foci. However, no differences in miR-21 expression between the eutopic endometrium of women with endometriosis and control eutopic endometrium, confirmed in the study by Haikalis et al. [78], suggest that the modification of miR-21 expression may be a secondary molecular change in the pathogenesis of endometrial implants. Nevertheless, the validity of miR-21 identification for the molecular mechanisms involved in the development of endometriosis is emphasized by the fact that miR-21-5p expression occurs at statistically significantly lower levels after endometriotic peritoneal fluid treatment of primary cell cultures from eutopic endometrial tissue obtained from patients, which allows to open up new therapeutic strategies in the treatment of endometriosis through the use of miR-21 [81]. It is also suggested that there are molecular differences in the profile of the miRNA studied by us between endometrial lesions collected from different anatomical sites. A comparison of the level of miR-21 expression between implants from the peritoneal cavity, deeply infiltrating endometriosis, and endometrial lesions of the ovary indicates a significantly higher expression in the last case [78]. Thus it should be pointed out that the miR-21 expression profile may be dependent on the location of an endometrial lesion.

Not without significance for the miR-21 expression pattern is also the degree of development of the endometrial focus and the fact that the miRNA is involved in angiogenesis [81] and controls cell growth, invasion and tissue repair, potentially by regulating gene silencing and epigenetic mechanisms [82]. Interestingly, in our study and in other works [83], the regulation of miR-21 has been noted in severe vs. mild endometriosis. Moreover, there is evidence that miR-21 inhibits the translation of *TGFβ-1* mRNA in various diseases, including endometriosis [81,82,84,85], and the post-transcriptional regulation of miR-21 via *SMAD3* plays a key role in its upregulation and the development of fibrosis [45].

Our study suggests that both miR-21 and the components of the TGF-β1-SMAD-ILK signaling pathway, to which it is targeted, are expressed differentially between ectopic endometrial lesions and eutopic endometrium in women with endometriosis. In this study, *TGF-β1*, *SMAD3* and *ILK* mRNA transcripts in endometrial foci were downregulated, and the level of miR-21 expression was significantly high compared to eutopic and control tissue. It has been proven that the inhibition of miR-21-5p effectively activates the apoptotic potential of endometrial cells, and the high level of expression of this miRNA may reflect the characteristics of endometriosis, such as the pathological growth or proliferation of cells [79]. Thus, we hypothesize that excessive suppression of the studied genes occurs through miR-21, especially inhibition of the anti-inflammatory cytokine *TGF-β1*, also known as the main promoter of EMT [14,15,52]. It is assumed to mediate a cascade of events from the transition from an initially ischemic and inflammatory environment, with the consequent tissue damage and necrosis, to an environment that promotes cellular proliferation, tissue remodeling and fibrosis during the development and progression of endometrial lesions, and the current findings partly confirm our assumptions.

The interesting results of our work are the negative correlations between miR-21 and the studied genes. They indicate that miR-21 mediates post-transcriptional regulation in peripheral blood mononuclear cells (PBMCs) in women with endometriosis and regulates transcripts in the studied molecular network associated with endometriosis, with the strongest correlation detected between miR-21 and *TGF-β1*, which confirms that this gene is a direct target for miR-21. Now there is a need to develop a non-invasive test for patients with symptomatic endometriosis; however, as of today, despite many biomarkers of endometriosis proposed in peripheral blood, none have been approved for endometriosis.

To the best of our knowledge, we were the first to assess changes in the level of expression of *TGF-β1, SMAD3, ILK* and miR-21 in the PBMC of patients with and without endometriosis. Such an approach seems very important because the ectopic foci of peritoneal endometrium provoke a local inflammatory response with the recruitment of macrophages, release of cytokines and production of reactive oxygen species [86], which may be reflected systemically. It can be expected that specific genes involved in leukocyte activation participate in the abnormal development of the immune response and are expressed differently in the blood leukocytes of women with endometriosis compared to the control subjects. Bearing in mind the role of an important immune suppressor such as *TGF-β1* in the activation of Th17 cells [87], as well as the release of that cytokine by the T-reg lymphocytes [88], the disturbance of the distribution and activation of the Treg and Th17 lymphocyte system in an inflammatory disease such as endometriosis may be related to the deregulation of *TGF-β1* signaling in peripheral blood. However, the results of our study show that the systemic gene levels of the TGF-β1-SMAD3-ILK pathway, as well as the miR-21 targeting this axis in women with endometriosis, did not change compared to women without the disease. We conclude that endometriotic cells have not changed the immunophenotype of peripheral lymphocytes, and the extent of the microenvironmental impact of endometrial foci is probably local and affects the peritoneal fluid, in which differentiated expression of anti-inflammatory cytokines, including *TGF-β1*, is observed in women with endometriosis [89].

In summary, the molecular cascade in the pathogenesis of endometriosis is still not fully understood. Nevertheless, it is generally accepted that variations in gene expression patterns and regulatory miRNAs in endometrial stromal cells are important factors in the development and maintenance of endometrial lesions. Based on our experimental results involving biological material (samples from the ectopic endometrium, its eutopic counterpart, and from the control endometrium), we found higher expression levels of the mRNA *TGF-β1*, *SMAD3*, and *ILK* in the eutopic endometrium and reduced expression levels in ectopic lesions compared to control tissue. In women with endometriosis, the miR-21 expression level in the eutopic endometrium was similar to the control but lower than in endometrial lesions. We also confirmed negative correlations between miR-21 expression levels and the studied genes, identifying this miRNA as a potential negative regulator of the TGF-β1-SMAD3-ILK axis. This finding indicates that genes studied by us and miR-21 may be some of the factors involved in the molecular pathogenesis of endometriosis. However, more research focused on this topic needs to be conducted for the accurate evaluation of the exact mechanism of molecular interactions between miR-21 and the TGF-β1-SMAD3-ILK signaling pathway in endometriosis.

Regarding the strengths and weaknesses of our study, we highlighted the importance of the miR-21-TGF-β1-SMAD3-ILK axis in the pathogenesis of endometriosis. The novelty of our work was the analysis of the expression of miR-21, *TGF-β1*, *SMAD3* and *ILK* in PBMCs obtained from women with diagnosed endometriosis compared to healthy ones in expectation of changes in the expression level profile of the studied genes/miRNA in connection with changes in the immune response (Treg and Th17 lymphocyte system). Unfortunately, we did not observe differences in the expression levels of miR-21, *TGF-β1*, *SMAD3* and *ILK* in PBMCs in women with and without the disease, which excluded indications for further immunophenotype assessment.

Our study has some limitations that need to be addressed. First, at this stage of our research, we were unable to perform experiments to verify the mechanism of action of miR-21 in the TGF-β1-SMAD3-ILK signaling pathway and the function of these molecules in EMT; second, the sample size of our study was limited. Therefore, in the future, we plan to analyze cell lines regarding the effect of miR-21, *TGF-β1*, *SMAD3*, and *ILK* expression levels on the biological functions of endometrial cells, which will provide innovative theoretical references in terms of molecular targets for endometriosis treatments.

## 4. Materials and Methods

### 4.1. Research Ethics

This study was conducted in accordance with Good Clinical Practice and the principles of the Helsinki Declaration. All methods were carried out in accordance with the relevant guidelines and regulations. The protocols of this study were approved by the Bioethics Committee of the Medical University in Lodz (resolution No. RNN/67/19/KE, 12 February 2019). All participants were fully informed and signed an individual consent form for participation in the study.

### 4.2. Clinical Groups and Sample Collection

**Figure 3:**
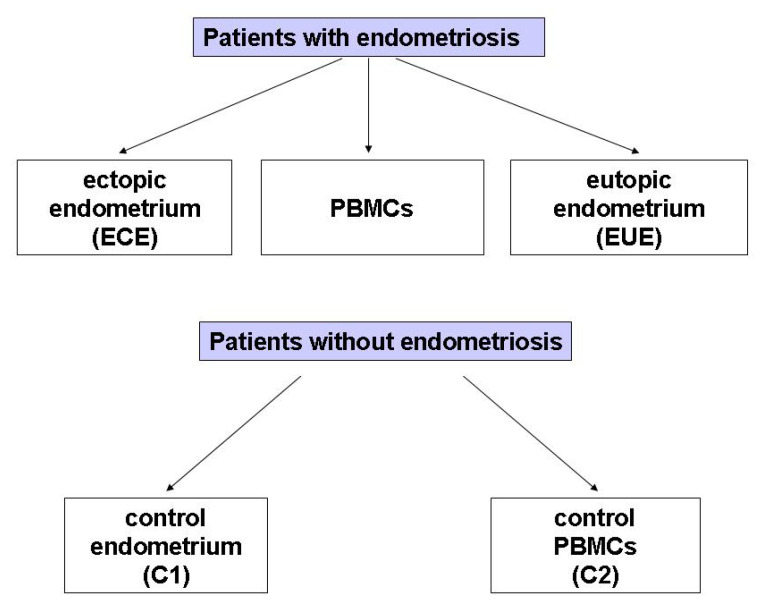
The groups of biological material obtained from patients with diagnosed endometriosis selected for the study.

The study included 29 women with endometriosis (mean age: 38.38, SD: 6.83 years; age range: 22–49 years) and 25 (mean age: 44.68, SD: 3.34 years; age range: 38–44 years) without endometriosis at the Operative and Conservative Gynecology Ward, Dr. K. Jonscher Municipal Medical Centre, Lodz, Poland, admitted in the period of March 2019–May 2020. The exclusion criteria for all participants were: pregnancy, breastfeeding, the presence of leiomyoma, adenomyosis, or cancer of the uterine and cervix, as well as of other diseases of the uterus, fallopian tubes and ovaries, and hormone treatment for at least 12 months at the time of surgery. The diagram illustrating the biological material obtained from patients with and without endometriosis selected for the study is presented in Figure 3.

Pathological confirmation was necessary for all patients. The inclusion criteria were: diagnosis of endometriosis with simultaneous surgical treatment. During the elective diagnostic or therapeutic laparoscopy for suspected extrauterine endometriosis due to pain symptoms and/or infertility investigation, biological material was obtained from the same patient from 3 locations (paired samples):

(1) A piece of tissue taken from an ovarian endometrial cyst (*n* = 27) or sections from the peritoneum of the small pelvis (*n* = 2); ectopic endometrium (ECE),

(2) A piece of the mucosa/endometrium from the uterine cavity obtained by biopsy (n = 25); eutopic endometrium (EUE); no biopsy was obtained from 4 patients with endometriosis,

(3) whole blood from which peripheral blood mononuclear cells (PBMCs) were isolated (*n* = 25). Whole blood was not obtained from 4 patients with endometriosis.

Paired EUE, ECE tissues and PBMCs were obtained from women who presented with mild to severe endometriosis (*n* = 25). The diagnosis of endometriosis for all obtained biological samples was confirmed by histopathological examination. According to the criteria laid down by the revised American Fertility Society guidelines [90], the investigated biological samples (*n* = 29) were divided into 4 groups according to the stage of endometriosis: I (*n* = 1), and II (*n* = 2), III (*n* = 16), and IV (*n* = 10). Additionally, women with endometriosis reported mild (*n* = 11), moderate (*n* = 2), or severe (*n* = 16) pelvic pain, which was distinguished using the numerical rating scale (NRS). Before surgery, the fertility status and concentration data of cancer antigen (CA)-125 and human epididymis protein 4 (HE4) were collected. The results of CA-125 were not available for 3 patients, and the results of HE4 were not available for 14 patients. The phases of the menstrual cycle were determined on the basis of the histological evaluation of the endometrium and the date of the patient’s last menstruation. Seven (7) patients were in the proliferative phase, and eighteen (18) were in the secretory phase of their cycle, which was divided into early (*n* = 7), middle (*n* = 4) and late (*n* = 7).

Control samples were collected from patients with uterine myomas who underwent hysteroscopy or laparoscopic myomectomy without visual evidence of endometriosis and adenomyosis. During the surgery, the absence of endometriosis and adenomyosis was confirmed by meticulous examination of the pelvic and extra-pelvic peritoneum, ovaries, intestine, and diaphragm. Histopathological evaluations confirmed normal endometrium in all controls. The patients in the control group did not suffer from endometriosis. Six (6) patients were in the proliferative phase, and nineteen (19) were in the secretory phase of the cycle, which was divided into early (*n* = 5), middle (*n* = 5) and late (*n* = 9). Paired samples were obtained from each woman, including:

(1) A piece of the normal endometrial tissue from the uterine cavity obtained by biopsy (*n* = 25); C1;

(2) Whole blood, from which PBMCs were isolated (*n* = 25); C2.

### 4.3. RNA Isolation from Eutopic, Ectopic, Normal Endometrial Tissue and Peripheral Blood Mononuclear Cells (PBMCs)

Patient and control tissue samples were placed in fixRNA buffer (Eurx, Gdańsk, Poland) and stored at 2–8 °C until use. After the fixRNA buffer was removed, the samples were divided into smaller portions with 600 μL of lysis buffer added and homogenized using a Pellet pestle homogenizer (Sigma-Aldrich, Darmstadt, Germany).

A total of 5 mL of whole blood was collected on EDTA from each of the patients and controls. In order to obtain PBMCs, the blood samples were centrifuged in a density gradient using the Histopaque-1077 agent (Sigma-Aldrich, Poznań, Polska) according to the manufacturer’s protocol. The peripheral blood mononuclear cells were placed in fixRNA buffer (Eurx, Gdańsk, Poland) and frozen at −80 °C until use.

Total RNA isolation from tissue homogenates and PBMCs was performed using the mirVana miRNA Isolation Kit (Life Technologies, Carlsbad, CA, USA) according to the manufacturer’s protocol.

Qualitative and quantitative evaluation of the isolated RNA was performed by the spectrophotometric method, measuring the absorbance with the Eppendorf BioPho-tometerTM Plus (Eppendorf, Hamburg, Germany) at 260/280 nm wavelengths. Only samples fulfilling the following requirements were selected for further use: miRNA concentrations of 1–10 ng/μL and RNA concentration over 50 ng/μL, with a 260/280 nm ratio of 1.8–2.0. Then, RNA was aliquoted and frozen at −80 °C until the real-time polymerase chain reaction (qPCR) was performed.

### 4.4. Reverse Transcription (RT) and Real-Time Quantitative Polymerase Chain Reactions (Real-Time qPCR)

The reverse transcription reaction for genes was performed using the High-Capacity cDNA Reverse Transcription Kit (Applied Biosystems, Carlsbad, CA, USA) in a volume of 20 μL. The reaction mixture contained: 10× RT buffer, 25× dNTP Mix (100 mM), 10× RT Random Primers, MultiScribe Reverse Transcriptase (50 U/µL), RNase Inhibitor and nuclease-free water. A total of 100 ng of total RNA was added to the reaction mixture. The following RT reaction conditions were used: 10 min at 25 °C, 120 min at 37 °C, 5 min at 85 °C, and cooling at 4 °C.

The reverse transcription for miRNA of 5 μL (10 ng) of total RNA in a 15-μL reaction was carried out using a TaqMan MicroRNA Reverse Transcription Kit (Applied Biosystems, Carlsbad, CA, USA). The RT master mix contained: 25× dNTP Mix (100 mM), MultiScribe Reverse Transcriptase (50 U/µL), 10× RT buffer, RNase Inhibitor (20 U/µL), and nuclease-free water, as well as the specific RT primers (small RNA-specific RT primers) included in individual TaqMan MicroRNA Assays: hsa-miR-21-5p (UAGCUUAUCAGACUGAUGUUGA) and RNU6B (CGCAAGGATGACACGCAAATTCGTGAAGCGTTCCATATTTTT) as endogenous control (Applied Biosystems, Carlsbad, CA, USA). The following RT reaction conditions were used: 30 min at 16 °C, followed by 30 min at 42 °C; then, the samples were heated to 85 °C for 5 min and held at 4 °C.

The negative control was included in each RT reaction using water instead of RNA. The RT reaction was performed in a Personal Thermocycler (Eppendorf, Germany). RT products were stored at −20° C until further analysis.

Relative genes/miRNA expression was assessed by real-time polymerase chain reaction (qPCR) using a 7900HT Fast Real-Time PCR System apparatus (Applied Biosystems, Carlsbad, CA, USA). A total reaction mixture volume of 20 μL contained: cDNA (1–100 ng), KAPA PROBE FAST qPCR Master Mix (2X) ABI Prism (Kapa Biosystems Ltd., London, UK), RNase-free water, and a 20xTaqMan Gene Expression Assay for the following genes: *TGF-β1* (Hs00998133_m1), *SMAD3* (Hs00969210_m1), *ILK* (Hs00177914_m1) and *GAPDH* (Hs99999905_m1), which was selected as the reference gene in the qPCR reaction. Assays for the following miRNA: miR-21-5p and RNU6B (endogenous control) were used in the qPCR reaction.

The relative expression levels of the analyzed gene/miRNA were evaluated by the delta-delta CT method (TaqMan Relative Quantification Assay software, Applied Biosystems) and presented as RQ values relative to the *GAPDH*/RNU6B reference genes/miRNA, respectively. The following formula was used to determine the ΔΔCT value: ΔΔCT = ΔCT test sample − ΔCT calibrator sample. For the calibrator (RNA isolated from biological material from a healthy patient without endometriosis), the RQ (relative quantification) value was considered equal to 1. In the case of the test samples, increased expression was recognized when the RQ value was more than 1, and decreased expression was recognized when the RQ value was less than 1.

### 4.5. Statistical Analysis

The statistical analysis was carried out using the Statistica 13.1 program (StatSoft, Cracow, Poland). The Shapiro–Wilk test showed that the data distribution was not normal. In order to search for the statistical significance between the study groups, Mann–Whitney U-test and/or Kruskal–Wallis tests were performed, depending on the size of the groups. Neuman–Keuls’ multiple comparison test was used to identify possible significant differences in RQ values between the individual variables. The Spearman rank correlation coefficient was used to measure the direction and strength of the relationship for individual variables and the potential relations between RQ genes and miRNA. The level of correlation was fixed in the following categories: very strong (Rho ≥ 0.80), moderate (Rho = 0.60–0.79), fair (Rho = 0.30–0.59) and poor (Rho ≤ 0.29) [91]. For all statistical analyses, the level of statistical significance was assumed at *p* < 0.05. The RQ values for the studied genes/miRNA are presented as mean.

## 5. Conclusions

The eutopic endometrium of women with endometriosis showed higher expression of the mRNA *TGF-β1*, *SMAD3* and *ILK*, and the level of miR-21 did not change compared to the endometrium of healthy participants. In contrast, lower mRNA levels of the studied genes and higher miR-21 levels were present in their endometrial implants. Our results may suggest a loss of endometrial epithelial phenotype defined by the differential expression of the *TGF-β1*, *SMAD3* and *ILK* genes in eutopic and ectopic endometrium, and the likely underlying mechanism may involve EMT via the TGF-β1-SMAD3-ILK signaling pathway. We also identified miR-21 as a possible inhibitor of that signaling (TGF-β1-SMAD3-ILK) in the context of endometriosis. Although functional research on the effects of disturbances in the expression pattern of the studied genes and miRNA in the disease mechanisms, especially in the induction/progression of endometrial EMT, as well as a comprehensive insight into this system, will require analysis at the protein level, these data, including previous evidence, suggest a role of *TGF-β1*, *SMAD3* and *ILK*, as well as the regulator miR-21, in the pathogenesis of endometriosis.

## Figures and Tables

**Table 1:** The expression levels (mean RQ values) of the studied genes and miR-21 in the PBMCs in the group of patients without endometriosis (C2) vs. the group of patients with endometriosis.

Patients	RQ (mean) in PBMCs			
	*TGF-ß1*	*SMAD3*	*ILK*	miR-21
Without endometriosis (C2)	0.259900	0.493684	0.319592	0.022728
With endometriosis	0.257616	0.520224	0.273768	0.026264
*p-value*	*0.500405*	*0.729190*	*0.182269*	*0.476172*

**Table 2:** Expression level (mean RQ value) of tested genes and miRNA in ectopic tissue in relation to clinical features and biochemical parameters of patients with endometriosis.

Features (*n*)	RQ (mean)			
	*TGF-ß1*	*SMAD3*	*ILK*	miR-21
Age: years ≤40 (18) >40 (11)	0.45747 0.25385	0.44777 0.27795	0.59452 0.27689	15.91973 16.65276
*p-value*	*0.220433*	*0.220433*	*0.083554*	*0.256430*
rASRM: III (16) IV (10)	0.35503 0.41274	0.42791 0.39368	0.50639 0.48345	14.50213 18.73967
*p-value*	*0.856355*	*0.134991*	*0.086857*	*0.622736*
Pelvic pain symptoms: mild (11) severe (16)	0.38048 0.35503	0.36190 0.42791	0.44356 0.50639	17.37284 14.50213
*p-value*	*0.576537*	*0.071229*	*0.044271* *	*0.450952*
Ca 125 (U/mL): ≤65 (10) >65 (16)	0.428850 0.344963	0.438240 0.400056	0.554670 0.461881	13.55235 17.74421
*p-value*	*0.897126*	*0.622736*	*0.516940*	*0.736605*
HE4 (pmol/l): ≤50 (8) >50 (7)	0.265788 0.522729	0.347087 0.551757	0.342725 0.681400	27.11661 13.46677
*p-value*	*0.866511*	*0.535820*	*0.189277*	*0.280963*
Cycle phase: Proliferative (7) Secretory (18): Early (7) Middle (4) Late (7)	0.365371 0.299228 0.247143 0.264800 0.370986	0.394786 0.289467 0.342629 0.378125 0.185643	0.461400 0.316883 0.325714 0.387075 0.267943	15.45009 18.41140 11.93261 20.80028 23.52511
*p-value*	*0.8884*	*0.0744*	*0.6994*	*0.7343*

* statistically significant *p* < 0.05. Abbreviation: rASRM: Revised American Society for Reproductive Medicine classification system for endometriosis.

## Data Availability

Data will be made available on request.
